# Coumarin Interferes with Polar Auxin Transport Altering Microtubule Cortical Array Organization in *Arabidopsis thaliana* (L.) Heynh. Root Apical Meristem

**DOI:** 10.3390/ijms22147305

**Published:** 2021-07-07

**Authors:** Leonardo Bruno, Emanuela Talarico, Luz Cabeiras-Freijanes, Maria Letizia Madeo, Antonella Muto, Marco Minervino, Luigi Lucini, Begoña Miras-Moreno, Adriano Sofo, Fabrizio Araniti

**Affiliations:** 1 Dipartimento di Biologia, Ecologia e Scienza della Terra, Università della Calabria (DiBEST-UNICAL), 87036 Arcavacata di Rende, Italy; emanuela.talarico@unical.it (E.T.); marialetizia.madeo@unical.it (M.L.M.); antonella.muto@unical.it (A.M.); marco.minervino@unical.it (M.M.); 2 Department of Plant Biology and Soil Science, Campus Lagoas-Marcosende, University of Vigo, 36310 Vigo, Spain; lcabeiras@uvigo.es; 3 CITACA, Agri-Food Research and Transfer Cluster, Campus da Auga, University of Vigo, 32004 Ourense, Spain; 4 Department for Sustainable Food Process, Università Cattolica del Sacro Cuore, Via Emilia Parmense 84, 29122 Piacenza, Italy; luigi.lucini@unicatt.it (L.L.); mariabegona.mirasmoreno@unicatt.it (B.M.-M.); 5 Department of European and Mediterranean Cultures: Architecture, Environment, and Cultural Heritage (DICEM), University of Basilicata, 75100 Matera, Italy; adriano.sofo@unibas.it; 6 Dipartimento di Scienze Agrarie e Ambientali—Produzione, Territorio, Agroenergia, Università Statale di Milano, Via Celoria n°2, 20133 Milano, Italy

**Keywords:** specialized metabolite, phytotoxic, lateral roots, root apical meristem, root swelling, cortical microtubules

## Abstract

Coumarin is a phytotoxic natural compound able to affect plant growth and development. Previous studies have demonstrated that this molecule at low concentrations (100 µM) can reduce primary root growth and stimulate lateral root formation, suggesting an auxin-like activity. In the present study, we evaluated coumarin’s effects (used at lateral root-stimulating concentrations) on the root apical meristem and polar auxin transport to identify its potential mode of action through a confocal microscopy approach. To achieve this goal, we used several *Arabidopsis thaliana* GFP transgenic lines (for polar auxin transport evaluation), immunolabeling techniques (for imaging cortical microtubules), and GC-MS analysis (for auxin quantification). The results highlighted that coumarin induced cyclin B accumulation, which altered the microtubule cortical array organization and, consequently, the root apical meristem architecture. Such alterations reduced the basipetal transport of auxin to the apical root apical meristem, inducing its accumulation in the maturation zone and stimulating lateral root formation.

## 1. Introduction

Because of sessile conditions, plants have evolved marked metabolic plasticity to increase their defense and competitive abilities. They have developed different biochemical pathways involved in the biosynthesis of a plethora of specialized metabolites to counteract the challenges arising during their growth and development [1]. These small molecules, characterized by an overwhelming structural diversity, a robust taxonomic restriction, and specificity within species, play a pivotal role in plant survival and adaptation to the environment. Plants could use them for a wide range of functions such as communication, reproductive purposes, nutrient acquisition, and trophic interactions [2,3]. For most known secondary metabolites, their specific ecological role, their involvement in physiological processes, and their mode of action have not yet been established [4].

The plant–plant interaction mediated by specialized metabolites is known as allelobiosis (positive interaction) and allelopathy (negative interaction), and the molecules at the basis of these interactions are known as allelochemicals. This ecological phenomenon plays an essential role in plant adaptation and competitive ability in the ecosystem, allowing plants to positively or negatively affect neighbouring species’ growth and development [5].

Some allelochemicals used by plants as a chemical weapon to increase their competitive abilities could represent promising alternatives for producing new potential eco-friendly herbicides [6]. Moreover, they could positively/negatively affect the growth and development of sensitive species interfering with several biochemical and physiological aspects shaping their morphology [7].

Coumarins constitute a widely studied class of allelochemicals produced through the phenylpropanoid pathway via o-hydroxycinnamic acid lactonization [8,9]. They are present in almost all higher plants and microorganisms and are actively released into the environment through root exudation or dead plant tissue decay [10,11]. These specialized metabolites are involved in several ecological roles, such as the modulation of dynamical processes of species coexistence in plant communities [12], Fe acquisition and assimilation through root hair exudation and re-absorption [13,14,15,16], and the shaping of root microbiome composition [17]. 

The simplest compound belonging to this chemical class is 1,2-benzopyrone, also known as coumarin, mostly known for its phytotoxicity and potential hormone-like activity [18,19]. One of the first hypotheses suggesting an auxin-like effect of coumarin was proposed by Neumann [20]. He demonstrated that this specialized metabolite stimulated the elongation of *Helianthus* hypocotyls’ excised segments, suggesting that its action was comparable to auxin. This hypothesis was further supported by Jansson and Svensson [21], who observed that coumarin alone causes an increase in fresh weight mainly by stimulating large numbers of roots and increasing soybean biomass. Abenavoli et al. [19], studying the effects of coumarin on the root morphology of *Arabidopsis thaliana*, observed that low concentrations (≃100 µM) of this molecule reduced primary root growth and stimulated the lateral root number parameter. Those findings strongly supported the hypothesis that coumarin could exert an auxin-like activity or interact with auxin distribution within the root. Successively, Lupini et al. [22], using several transgenic lines of *Arabidopsis thaliana* (defective in influx and efflux carriers), demonstrated that the efflux carrier PIN2 and the influx carrier AUX1 could be involved in coumarin-induced root branching, suggesting that auxin redistribution might be directly or indirectly affected by this molecule.

However, the exact mechanism of coumarin on root growth has not been clarified yet. A complex molecular signalling network probably governs coumarin’s morpho-physiological responses, where auxin transport and/or biosynthesis could play an important role. In fact, auxin is considered the leading candidate in controlling the stress-induced morphogenic response inducing the inhibition of root elongation, enhancing lateral roots formation and stimulating the production of adventitious roots under stressful environmental conditions [23,24]. Its polar transport plays a central role in organ development and elongation, in shoot/root branching and plastic growth responses [25], in lateral root initiation [26,27], in lateral root primordial formation [28], and in emergence [29]. Transported via influx and efflux proteins in a polarized stream, auxin action depends on its differential distribution along with tissues [28,30], and it is known that its fluxes are driven by an interplay between cell wall structure and the dynamics of microtubule and actin filaments [31]. Moreover, it has been demonstrated that intact microtubules are required for polar auxin trafficking [32].

Recently, it has been demonstrated that several natural compounds exert their biological activity interfering with auxin distribution along the root and its biosynthesis [33,34,35,36,37,38,39,40]. Moreover, it has been proven that compounds such as citral, farnesene, norharmane, weisiensin B, and narciclasine strongly affect the microtubule organization, altering the root ultrastructure of the *Arabidosis* root, as well as its isotropic growth [33,36,37,41,42]. As two of the main effects exerted by coumarin are lateral roots formation and root tip swelling (a phenomenon observed with several microtubule interferents) [43], and the maintenance of auxin fluxes is known to depend on the interaction between cytoskeleton and PINs proteins [31], we hypothesized that the main coumarin effects could be related to microtubule alteration, followed by an alteration in PIN distribution. Therefore, using sub-lethal doses of coumarin, we decided to investigate coumarin’s effects on the primary root tip anatomy, microtubule organization, and polar auxin transport, trying to deepen our knowledge on the potential mode of action of this specialized metabolite.

## 2. Results

### 2.1. Effects of Coumarin on RAM

**Figure 1:**
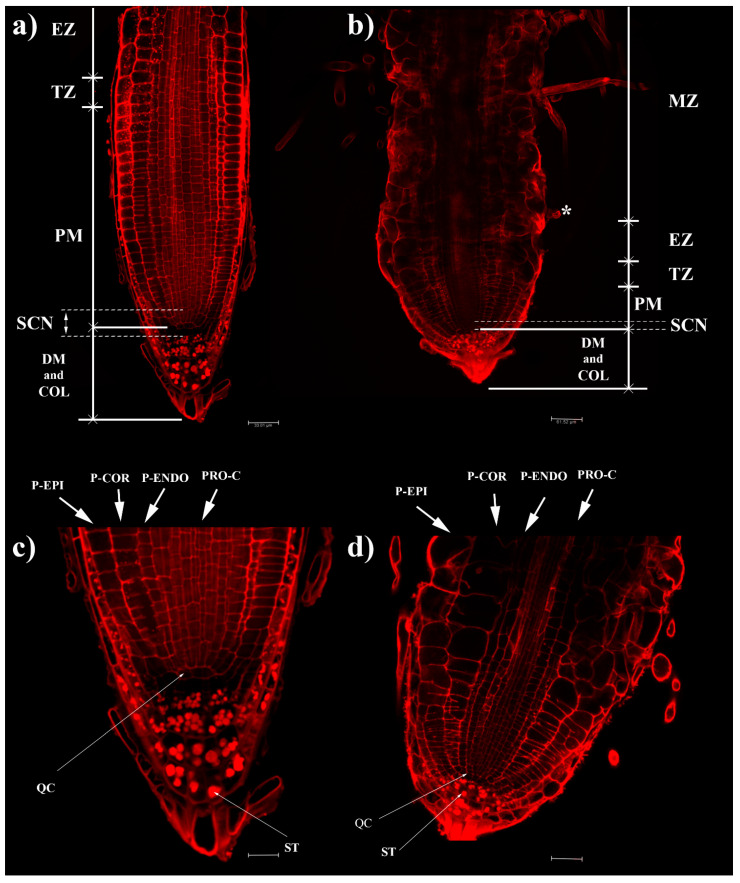
(**a**–**d**) Confocal laser microscope images of primary root tip, stained with propidium iodide, in 4 days old seedlings of *A. thaliana* treated for 7 days with coumarin 0 (**a**,**c**) and 100 μM (**b**,**d**). (**c**,**d**) Higher magnification of (**a**,**b**), respectively. *Arabidosis* RAM developmental zones: meristematic zone (MZ), the transition zone (TZ), and the elongation zone (EZ). The meristematic zone is divided into the distal meristem (DM) and the proximal meristem (PM). Moreover, in RAM, the stem cell niche (SCN) could be observed, as well as the quiescent center (QC), statoliths (ST), protodermis (P-EPI), precortex (P-COR), proendodermis (P-ENDO), and procambium (PRO-C); * first visible root hair. Scale bars: (**a**) 33.01; (**b**) 61.52; (**c**) 66.02; (**d**) 123.04 μm. N = 20.

**Figure 2:**
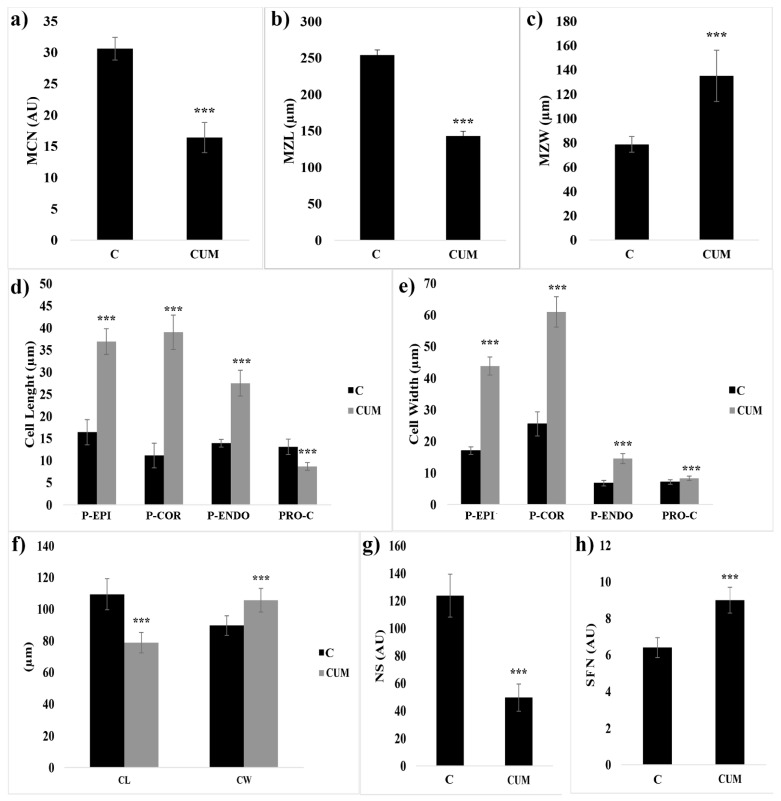
RAM morphology of 4 days old *Arabidosis* seedlings treated for 7 days with coumarin 0 (C) or 100 (CUM) µM. (**a**) MCM—meristem cell number; (**b**) MZL—meristem zone length; (**c**) MZW—meristem zone width; (**d**) cell length; (**e**) cell width; (**f**) CL—columella length; CW—columella width; (**g**) NS—number of statoliths; (**h**) SFN—procambium files number. Protodermis (P-EPI); precortex (P-COR); proendodermis (P-ENDO); procambium (PRO-C). Data are presented as mean ± standard deviation (SD). Statistical analysis was performed using the Student’s *t*-test with *** *p* ≤ 0.001. N = 20.

Seven days of coumarin treatment significantly altered the RAM of *Arabidosis* (Figure 1 and Figure 2). In particular, coumarin-treated roots were characterized by a reduction in MCN (46% lower than that in control) (Figure 2a), accompanied by a decrease in MZL (44%) (Figure 2b) and an increase in MZW (1.2-fold higher than that in control) (Figure 2c). In coumarin-treated seedlings, these data suggest the advancement of the transition zone and a premature exit of cells from the meristematic area (Figure 1 and Figure 2).

The advancement of the transition zone in coumarin-treated roots was accompanied by an abnormal shape and asymmetric organization of protodermis and precortex cells characterized by a swollen and abnormal shape (Figure 1a–d). Therefore, we decided to quantify these alterations by measuring protodermis, precortex, proendodermis, and procambium cells at the first elongated precortex cell level.

In coumarin-treated seedlings, the protodermis, precortex, and proendodermis cells’ length and width were significantly increased by the treatment (Figure 1 and Figure 2d,e). On the contrary, the length (34%) and width (16%) of procambium cells were significantly reduced and increased, respectively (Figure 2d,e). On the contrary, the number of cell files forming the procambium was significantly higher (40%) than that in control (Figure 2h). Besides, a substantial alteration at the columella level was also observed. The columella of the *Arabidosis*-treated roots was characterized, compared to the control, by a reduction in CL (28%) and an increase in CW (18%) (Figure 2f). In addition, the number of statoliths was significantly reduced by the treatment (60% lower than that of the control) (Figure 2g), but no changes in columella stem cell layers were observed (data not shown). Other significant alterations in treated roots, such as incomplete cell division, fused lateral roots, high production of root hairs close to the meristem, and a high production of adventitious root primordia, were observed (Figure S1a–d).

**Figure 3:**
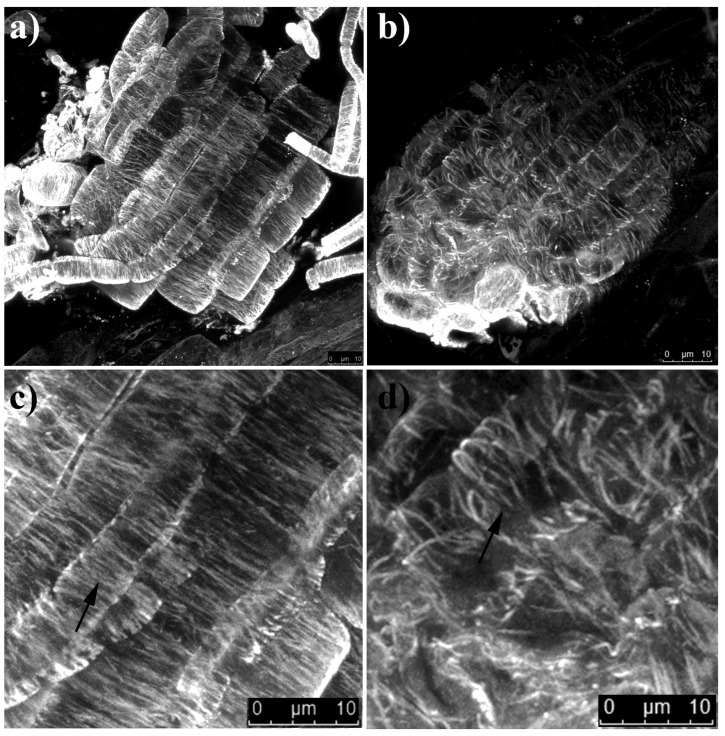
Microtubule immunostaining of 4 days old *Arabidopsis* roots treated for 7 days with coumarin. (**a**) Control cells (0 µM); (**b**) coumarin-treated cells (100 µM); (**c**,**d**) zoomed-in view of a specific area of the images of (**a**,**b**), respectively, to make coumarin-induced microtubule alterations clearly visible. Black arrows (in (**c**,**d**)) indicate parallel, dense, and well-organized microtubules in the control cells and disorganized and lax microtubules in coumarin-treated cells. Scale bars (images **a**,**b**): 10 μm. N = 20.

Concerning cortical microtubule immunolabeling, in control roots, microtubules were well-defined and typically arranged, parallel to the transverse axis of the cells, and uniform in density (Figure 3a,c). On the contrary, in treated roots, they were erratically arranged with evident loss of symmetry, reduced density, and microtubule strands thicker than those of the control (Figure 3b,d).

Finally, the alterations observed on colchicine-treated roots are similar to those of seedlings treated with coumarin, confirming its potential effect as a microtubule effector (Figure S2).

### 2.2. Effects of Coumarin on Cell Division

Given that root-meristem size is determined by both the rate of cell differentiation and cell division rate [44,45,46], to assess whether a reduction in cell division could also cause the decline in RAM size, we analyzed either the mitotic index or cyclin B’s turnover, which is an essential protein expressed during the G2 phase of the cell cycle, which is degraded during mitosis [47,48].

**Figure 4:**
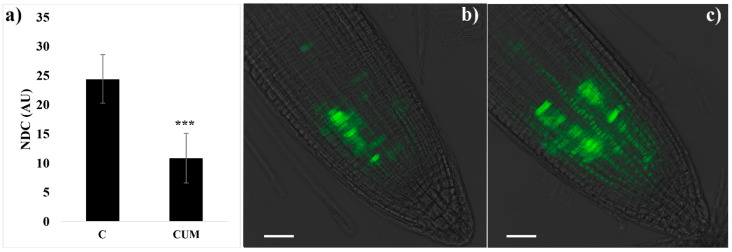
Effects of coumarin 0 (C) or 100 (CUM) µM on cell division in 4 days old *Arabidopsis thaliana* RAM. (**a**) Mitotic index expressed as the number of dividing cells (NDC). Data are presented as mean ± standard deviation (SD). Statistical analysis was performed using the Student’s *t*-test with *** *p* ≤ 0.001. N = 20. (**b**,**c**) Confocal microscopy imaging of *Arabidosis* transgenic line *cyclin B1;1::GFP* in control (**b**) and treated (**c**) RAM. Scalebar 50 µm.

The results reported highlighted a substantial reduction in dividing cells (55% lower than that of the control) (Figure 4a). The confocal microscopic analysis revealed that untreated *Arabidosis* seedlings showed a GFP-fusion cyclin B1;1 signal in some RAM cells (Figure 4b). In contrast, both the percentage of GFP-labeled cells and the relative intensity of the fluorescent signal slightly increased in roots treated with 100 µM of coumarin (Figure 4c). Those results suggest a disturbance in cyclin B’s degradation, resulting in abnormal mitosis in the RAM.

### 2.3. Effects of Coumarin on Auxin Content and Polar Transport

Previous research has suggested that the alterations observed on the root morphology of coumarin-treated (100 µM) roots could be mediated by an alteration in the polar auxin transport [22]. Therefore, we decided to quantify the auxin content in the roots of seedlings exposed to coumarin for 48 h and monitor its effects on the principal auxin transporting proteins.

Auxin distribution and quantification were carried out on the full seedlings roots using the auxin-responsive reporter *pDR5::GFP* and through a GC/MS approach. In contrast, coumarin’s effects on auxin transporting proteins were evaluated using the previously described GFP transgenic lines (see Materials and Methods).

**Figure 5:**
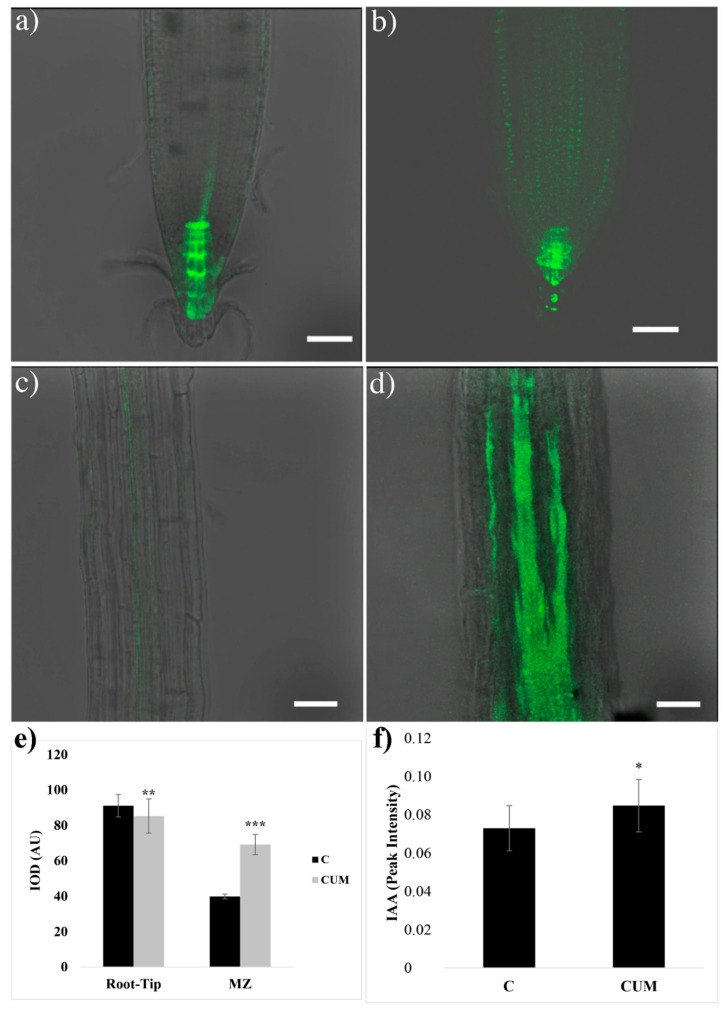
Fluorescence detection in 4 days old *A. thaliana pDR5::GFP* transgenic line (auxin responsive reporter) grown for 48 h in untreated agar medium ((**a**) (RAM) and (**c**) (elongation zone)) or 100 μM of coumarin enriched agar medium ((**b**) (RAM) and (**d**) (elongation zone)). Scale bars 34 μm. N = 20. (**e**) Integrated optical density (IOD) expressed as arbitrary units (AU) of fluorescence intensity measured in the root tip and the MZ (maturation zone) of *Arabidosis* treated for 48 h with 0 or 100 μM of coumarin (N = 10); (**f**) auxin content in *Arabidosis* roots treated for 48 h with coumarin 0 (C) or 100 (CUM) μM. Data are presented as mean ± standard deviation (SD). Statistical analysis was performed using the Student’s *t*-test with * *p* ≤ 0.05, ** *p* ≤ 0.01, *** *p* ≤ 0.001. N = 4.

The monitoring of the auxin-responsive reporter *pDR5::GFP* revealed that the 48 h coumarin treatment impaired auxin distribution. Indeed, in control roots, the *pDR5*-dependent GFP distribution reflected the typical auxin maximum distribution in the root tip (i.e., QC, initial and mature columella cells) and procambium cells (Figure 5). In coumarin-treated roots, a significant weak decrease (7%) in GFP signal intensity was observed in the root tip (Figure 5a,b,e). On the contrary, in the procambium at the MZ level, an increase in GFP intensity (73% higher than that in the control) was observed (Figure 5c–e). Besides, an accumulation of GFP fluorescence (higher than that in control roots (Figure 5c) was observed locally in the elongation zone and along the entire root until the root and stem boundary, suggesting auxin accumulation (Figure 5d).

To verify the effects of long coumarin exposure on DR5 signal, we also carried out experiments on plants treated for 7 days, which confirmed a substantial impairment in GFP signal intensity (Figure S3a,b). In the columella of the coumarin-treated root, the GFP was widespread, whereas in the procambium we observed a signal higher than that in control, confirming what was observed after 48 h of treatment (Figure S3b).

Finally, the GC/MS auxin quantification pointed out, in 48 h coumarin-treated roots, an auxin accumulation (16%) higher than that in control (Figure 5f).

**Figure 6:**
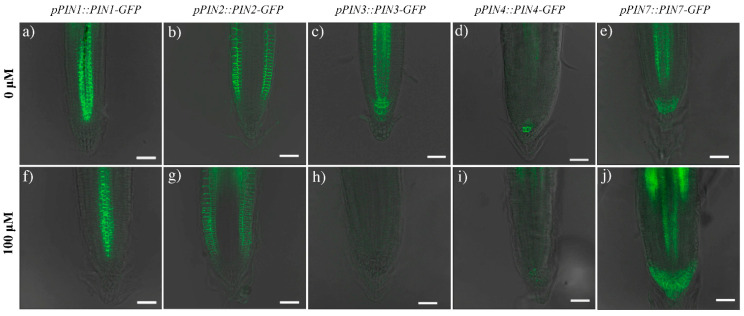
Images of the RAM in 4 days old seedlings of *A. thaliana* transgenic lines (*pPIN1::PIN1-GFP* (**a**,**f**), *pPIN2::PIN2-GFP* (**b**,**g**), *pPIN3::PIN3-GFP* (**c**,**h**), *pPIN4::PIN4-GFP* (**d**,**i**), *pPIN7::PIN7-GFP* (**e**,**j**)) treated for 48 h with coumarin 0 (**a**–**e**) or 100 μM (**f**–**j**). Scale bars 30 μm. N = 20.

Based on these observations, *PINs::PINs-GFP* (auxin efflux carriers) transgenic lines were used to monitor the location of the auxin transporters in coumarin-treated and untreated root tips (Figure 6a–j). Interestingly, the distribution pattern of three PIN proteins appeared strongly affected by coumarin treatment, showing an altered distribution, as well as a reduction in their presence.

**Figure 7:**
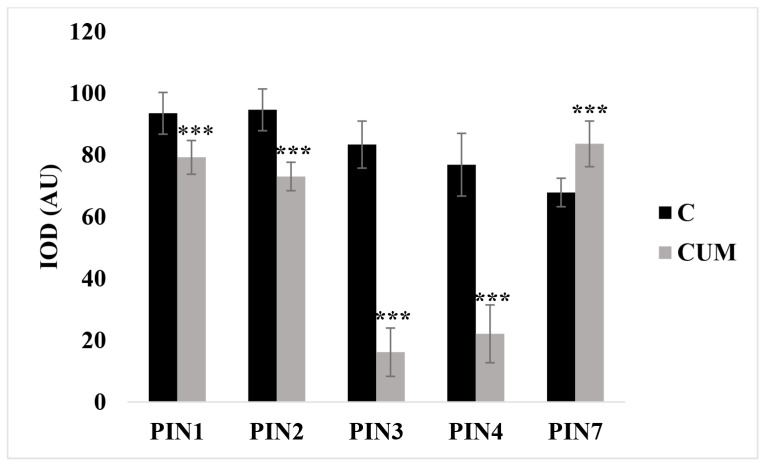
Integrated optical density (IOD) of PINs proteins expressed as arbitrary units (AU) of fluorescence intensity measured on the entire RAM of *Arabidosis* treated for 48 h with coumarin 0 (C) or 100 (CUM) μM. Data are presented as mean ± standard deviation (SD). Statistical analysis was performed using the Student’s *t*-test with *** *p* ≤ 0.001. N = 10.

In untreated roots, PINs proteins were characterized by a classical presence and distribution. As detailed in the bibliography [28], PIN1 is mainly situated at the basal end of provascular stelar and proendodermal cells (Figure 6a). PIN2 is localized basally in the procambium cells, the protodermal cells’ apical side, and the lateral root cap (Figure 6b). PIN3 is expressed in the columella, at the basal side of vasculature cells, and at the lateral side of the pericycle cells of the elongation zone (Figure 6c). PIN4 is localized in the stem cell niche and basally in provascular cells (Figure 6d). Finally, PIN7 resides at the lateral and basal membranes of provascular cells in the meristem and elongation zone (Figure 6e). In contrast, in columella’ cells, it coincides with the PIN3 domain [49] (Figure 6c,e). In plants treated with coumarin, no significant differences were observed in the PIN1 and PIN2 distribution along the RAM after 48 h of coumarin treatment. The GFP intensity was reduced by 16% and 32%, respectively (Figure 6a,b,f,g and Figure 7). The GFP fluorescence in PIN3 (IOD 81% lower than that in control) and PIN4 (IOD 72% lower than that in control) was absent (Figure 6c,d,h,i and Figure 7), whereas in PIN7, the GFP signal was highly intense in the basal side of the procambium cells and widespread in columella’s cells (Figure 6e,j), pointing out an increase in IOD that was 23% higher than that in control (Figure 7).

## 3. Discussion

In recent years, to reveal the effects of natural compounds on phytohormone pathways and their interference on plant growth and development, the model plant *A. thaliana* and its various mutants and transgenic lines have been widely employed.

In the present study, we evaluated coumarin’s effects on auxin biosynthesis and distribution by testing its effects on the root development of WT *Arabidosis* seedlings. Moreover, we assessed this molecule’s possible interaction with microtubule organization and its potential impact on auxin transport.

Coumarin’s phytotoxicity and its ability to alter both root anatomy and morphology have been primarily documented [12,50]. Previous studies have demonstrated that this molecule is a potent inhibitor of germination and seedling growth and development [51,52]. On the contrary, at relatively low concentrations (100 µM), it significantly stimulates lateral root and root hair production, whereas the total root length is significantly reduced [22]. Similar effects (reduction in primary root growth, increase in lateral root number, and root hair density) have generally been observed on *Arabidosis* seedlings treated with exogenous auxins such as IAA, 2,4-D, and NAA [53]. Due to that, it has been widely speculated that coumarin at low concentrations could exert an auxin-like effect [18,20], and recent studies have reported that coumarin’s effects are probably due to an alteration in the polar auxin transport [22]. In particular, Lupini et al. [22], using several auxin influxes and efflux mutants (*aux1-22*, *lax3*, *pin1*, *eir1-4*, and *pin3-5*), hypothesized that coumarin treatment (100 µM) could modulate root development interacting with the polar auxin transport. However, no further studies have been carried out to explore this potential mode of action in depth.

In this study, we demonstrated that coumarin’s effect is anything but an effect similar to auxin. The alterations induced by this molecule are, according to our data, a substantial alteration in the root tips cortical microtubule organization accompanied by an alteration in cell division and auxin transport, synthesis, and its *PINs*-mediated distribution.

Coumarin treatments induced a reduction in cell division and alteration in RAM organization. The RAM of treated plants was characterized by swollen protodermal and procambium cells, which expanded radially more than longitudinally. Moreover, a reduction in the RAM (formed by a lower number of cells than that in control) and an increase in its width were observed, suggesting an advancement of transition and differentiation zones. The reduction in meristem size is commonly observed in plants treated with natural products, heavy metals, and xenobiotics [40,54]. For example, a reduction in RAM size was observed in *Arabidosis* seedlings treated with brassinolide, farnesene, and narciclasine [40,44,55]. Besides, in plants treated with cadmium, early differentiation of meristematic cells was induced by a premature cell cycle exit from the meristematic zone [50].

In coumarin-treated seedlings, the reduction in meristem size was accompanied by an accumulation of CYCLIN B1;1, suggesting that coumarin could have affected the meristematic activity, resulting in the loss of cell division potential in the root. Previous studies have demonstrated that coumarin negatively affects mitosis in almost all phases, inducing chromosomal aberrations, binucleated cells, incomplete phragmoplasts, and DNA damages [56,57]. Culligan et al. [58] reported that DNA damaging agents could induce, as also observed in our experiments, the accumulation of CYCLIN B1;1, as well as root swelling. Moreover, Wu et al. [59], using the *Arabidosis* mutant *radially swollen 4* (*RSW4*) (a mutant with swollen root due to microtubule disorganization), suggested that CYCLIN B1;1 accumulates in response to DNA damages [60] or DNA damaging agents [58,61]. Besides, they observed that the superabundant cyclin is associated with altered root histology and root swelling mediated by microtubule disorganization. The hypothesis that CYCLIN B1;1 accumulation could induce an alteration in the microtubule cortical array organization was further confirmed by Weingartner et al. [62] in transgenic tobacco expressing a nondegradable version of CYCLIN B1;1 and by Serralbo et al. [63] using the *hobbit* mutant of *A. thaliana* characterized by a reduced function of the complex responsible for CYCLIN B1;1 degradation. Both mutant and transgenic lines were characterized by microtubule disorganization and swollen cell shape, supporting the idea that CYCLIN B1;1 accumulation disrupts cortical microtubules. Despite the evidence that CYCLIN B1;1 accumulation could alter microtubule organization, the possibility that these effects are directly induced by coumarin and not a consequence of pleiotropic effects at the moment is just speculation, which should be explored in depth.

The swelling phenomenon, generally accompanied by a reduction in RAM length, has mainly been observed in plants treated with microtubule interferents (stabilizers and destabilizers) such as colchicine, taxol, and oryzalin [64,65]. In particular, colchicine interferes with microtubule dynamics, blocking polymerization at the end of the mitotic spindle, leading to metaphase arrest [66]. In contrast, oryzalin binds to plant tubulin, preventing its polymerization and making extant microtubules more likely to depolymerize [64,67,68]. The effects of both molecules on root tip anatomy are similar to the effects induced by coumarin. The images of *Arabidosis* root tips presented by Baskin et al. [65] perfectly overlap with the effects observed in coumarin-treated plants. Those results suggest that coumarin might interact with the organization of microtubule arrays. The hypothesis was further confirmed by the immunolabeling bioassay, where microtubules of treated plants appeared fragmented and erratically arranged.

As the reduction in cell division and primary root growth, the increment in lateral root number, and the increase in root hair length and density are typical effects of plants treated with auxin and auxinic herbicides [69,70,71], we decided to investigate the effects of coumarin on both auxin content and transport. Moreover, Li et al. [72], using the hydroxycoumarin 4-methyl-umbelliferone, observed an auxin accumulation in *Arabidosis*-treated root that mediated F-actin disruption and, as a consequence, malformation of the RAM. Furthermore, it is known that auxin plays a pivotal role in the maintenance of root distal stem cell identity, and alterations in its balance could result in the loss of the QC identity, as well as an alteration in the cellular organization of the RAM in *Arabidosis* [73]. The GC-MS-driven relative quantification of auxin content pointed out a slight increase in this plant hormone in treated roots. This increase could justify the previously reported coumarin-induced stimulation of peroxidase and IAA-oxidase activity (enzymes involved in IAA catabolism) [74]. As well as coumarin, scopoletin (a natural coumarin with auxin-like activity and a chemical structure remarkably similar to the simple coumarin [7] at low concentrations inhibited auxin’s catabolism, causing its accumulation [75]. Simultaneously, as coumarin, it has been proven that the same molecule stimulated IAA oxidase activity [75]. Both molecules probably promote auxin accumulation, which activates IAA-oxidase activity to restore its concentration to physiological levels.

The auxin gene reporter *DR5::GFP* further suggested that coumarin also induced an alteration in its distribution as, in treated plants, a high *GFP* signal was observed in the elongation and maturation zone, as well as a weak reduction in the RAM. Casimiro et al. [76] demonstrated that auxin accumulation in the maturation zone is pivotal for lateral root production. They showed that the acropetal polar transport inhibitor NPA (N-1-naphthylphthalamic acid) induced IAA accumulation in the root apex and its reduction in basal tissues critical for lateral root initiation. They concluded that both root and shoot and root acropetal and basipetal auxin transport activities are required during the initiation and emergence phases of lateral root development. In addition, De Rybel et al. [77], using the synthetic non-auxin probe naxillin, a molecule that stimulates lateral root formation, reported (as also observed in our experiments) that 24 h of treatment with this molecule induced both the synthetic auxin-responsive marker *pDR5::GUS* locally in the maturation zone (specifically in xylem pole cells adjacent to the pericycle) and CYCLIN B1;1. They also demonstrated that the naxillin-auxin accumulation in roots was due to the naxillin-induced conversion of the auxin precursor indole-3-butyric acid into the active auxin indole-3-acetic acid. Besides, it has also been demonstrated that the induction of CYCLIN B1;1 expression coincides with the formation of a new lateral root primordium and, thus, lateral root development [78,79]. These results suggest that coumarin could alter auxin’s polar transport and synthesis by inducing auxin accumulation in the elongation and maturation zone of the root, activating the physiological and biochemical mechanisms involved in lateral roots production.

Previous studies focusing on the effects of microtubule de-polymerizing agents demonstrated that these molecules could interfere with auxin polar efflux transporters. In particular, oryzalin visibly interfered with PIN1 (in the procambium) and PIN2 (in young cortex cells), resulting in reduced auxin’s polar distribution. These findings indicated that intact microtubules are pivotal for proper auxin polar trafficking in plant cells [32]. In contrast to what was observed with oryzalin, coumarin did not affect the distribution of PIN1 and PIN2, but significantly reduced the protein abundance. On the contrary, it completely suppressed PIN3 and PIN4 protein abundance. Those results suggest that coumarin specifically inhibited the acropetal auxin flux in the procambium, in the columella cells (PIN3), and from the central RAM toward the quiescent center (PIN4). Therefore, auxin procurement to the RAM was only guarded by PIN1 and PIN7, which justifies the presence (even if lower than that in the control) of auxin in the root tip. The alteration in auxin balance observed at the quiescent center level could confirm our hypothesis that coumarin could induce the loss of the root distal stem cell identity and could justify the lack of columella cell expansion, resulting in a reduced columella area in treated roots. Moreover, the alteration in the local auxin maximum/gradient within the RAM, which is generated by the PIN directional auxin transporters, could be the reason for the reduction in statolith observed in coumarin-treated roots. In fact, Zhang et al. [80] demonstrated that auxin regulates the expression of three key starch granule synthesis genes involved in statolith production. Finally, the diffuse GFP signal observed in PIN7 in the elongation zone, as confirmed by the DR5 auxin reporter, suggests auxin accumulation in the elongation zone and particularly in the maturation zone of the root, where lateral root initiation occurs. It is known that auxin regulates root system architecture by promoting the acquisition of founder cell identity in pericycle cells [81,82] and by stimulating LR development [76,83].

## 4. Materials and Methods

### 4.1. Reagents Used

All the reagents used in the following experiments (EtOH, NaCLO, MES, Triton X-100, plant agar, propidium iodide, Schiff reagent, among others) were purchased from Sigma Aldrich S.r.l. (Via Monte Rosa, Milano, Italy).

### 4.2. Plant Material and Growth Conditions

Seeds of *Arabidopsis thaliana* (L.) Heynh. Ecotype Columbia (Col-0) and *pDR5::GFP* [28], *pPIN1::PIN1-GFP* [84], *pPIN2::PIN2-GFP* [49], *pPIN3::PIN3-GFP* [49], *pPIN4::PIN4-GFP* [49], *pPIN7::PIN7-GFP* [49], and *cyclin B1;1::GFP* [85] transgenic lines were used for the experiments. Seed sterilizations, growth conditions, and treatments were carried out as previously described by Lupini et al. [22], with some modifications. 

Seeds were surface-sterilized for 3 min in 50% EtOH, and they were then washed for 3 min in a NaOCl solution (0.5%) enriched with Triton X-100 at 0.01%. Finally, sterilized seeds were washed three times in sterilized Milli-Q water and, to break dormancy and synchronize germination, were then maintained in a 0.1% agar solution at 4 °C for 48 h. Twenty-four seeds were then placed in square Petri dishes (100 × 150 mm) containing agarised (0.8% *w*/*v*) basal medium enriched with micro and macronutrients, plus sucrose (0.5% (*w*/*v*)), MES (1 g L^−1^), pH 5.75 [22].

Petri dishes were placed vertically in a growth chamber at 22 °C, 65% relative humidity, 16 (light)/8 (dark) h photoperiod, and 300 µmol photon flux density m^−2^ s^−1^. Five 4 days old seedlings (per treatment and replicate), chosen by uniformity were then transplanted for seven days (long-term experiment) or 48 h (short-term experiment) in the agar medium previously described enriched with coumarin 0 (control) and 100 μM of coumarin (treatment).

### 4.3. Long-Term Experiments

To highlight drastic changes induced by coumarin on the *Arabidosis* root apical meristem (RAM) and cortical microtubule organization, otherwise not visible during short-treatments, 4 days old *Arabidosis* seedlings were treated with coumarin for 7 days.

#### 4.3.1. Root Anatomy: Meristem Size Analysis

For the analysis of the RAM, *A. thaliana* (Col-0) seedlings, for each treatment (coumarin 0 or 100 µM) and replication (N = 20), were fixed, stained with propidium iodide, and imaged by confocal microscopy according to Gonzàlez-Garcìa et al. [86].

At the end of the experiment, the following parameters were evaluated: (i) the number of cells composing the RAM (MCN), calculated counting the number of precortex cells extending from the quiescent center (QC) to the first elongated cortex cell; (ii) Meristem length (MZL), expressed as the distance (µm) from the QC to the first elongated cortex cell; (iii) RAM width (MZW), considered the distance between the two lines of cortex cells; (iv) the length and width of protodermal, precortical, and proendodermal cells; (v) the length (CL) (measured from the QC to the bottom of the columella) and width (CW) (measured at the QC level) of the columella; (vi) the columella (CA) area, considered as the total area of cells presenting statoliths.

A parallel experiment using colchicine (200 µM) was carried out to compare the effects of coumarin with a known microtubule polymerizing agent.

#### 4.3.2. Microtubules Immunolabeling

According to the method of Holzinger et al. [87], the microtubule immunolabeling was carried out with some modifications. After coumarin treatment (0 or 100 μM), *A. thaliana* root tips were excised and fixed at room temperature for 45 min in PEM buffer (50 mM of PIPES, 2 mM of EGTA, 2 mM of MnSO_4_; pH 7.2) containing 0.5% glutaraldehyde, 1.5% formaldehyde, and 0.1% Triton X-100. 

Roots were successively washed for 20 min in the same buffer and then again in PEM buffer. Fixed roots were chopped into small pieces and digested, at room temperature for 30 min, with cellulase and pectolyase Y-23 (1%) solubilized in PEM buffer (pH 5.5). After the digestion step, samples were washed in PEM buffer (pH 7.2) and incubated for 10 min at −20 °C in methanol. The samples were successfully washed with phosphate buffer saline (PBS) (pH 7.4) and incubated for 20 min with 1 mg mL^−1^ of sodium borate in PBS. Samples were then further washed with PBS and incubated with 1% bovine serum albumin and 50 mM of glycine in PBS. After a final washing in PBS, the samples were incubated overnight with the primary antibody (anti-α tubulin B 5-1-2, Sigma-Aldrich, 1:1000 in PBS) at 4 °C, which was removed by three consecutive washes in PBS. The samples were then incubated with the secondary antibody (Alexa 488-conjugated goat anti-mouse IgG, Sigma-Aldrich, 1:200 in PBS) at 37 °C for 3 h. Besides, negative controls were performed alternately using the primary and secondary antibodies alone (data not shown). 

Finally, immunolabelled roots were mounted in the Citifluor AF1 antifade agent to avoid fluorescence degradation. Visualization of the microtubules was performed using a Leica TCS SP5 confocal microscope (Wetzlar, Germany) with a 63X oil immersion objective and a 496 nm excitation wavelength (argon laser); and they were photographed with a LAS-AF software. The experiments were carried out with 20 replicates.

### 4.4. Short-Term Experiments

To avoid biased interpretation, due to cascade effects connected to prolonged exposure to the molecule, the experiments on cellular division, *PINs* proteins, and auxin quantification were carried out using a short exposure (48 h) of the seedlings to coumarin**.**

#### 4.4.1. Mitotic Indices and Cyclin B1;1::GFP Localization

Cell division was observed by confocal microscopy (see the method described below in § localization of GFP signal in *Arabidopsis*’s primary roots) on the *cyclin B1;1::GFP* transgenic line treated for 48 h on the previously described agarized medium enriched with coumarin 0 or 100 µM.

The evaluation of mitotic indices in *A. thaliana* RAMs treated for 48 h with coumarin 0 or 100 µM was carried out as previously described by Cools et al. [88] with some modifications. In particular, the synchronization of root cell division was obtained by incubating the seedlings for 24 h in a hydroxyurea solution (2 mM, pH 6.0). After incubation, *Arabidosis* seedlings were washed in deionized water and transferred in a continuously aerated coumarin solution (0 or 100 µM) for 48 h. Seedlings were then collected, and RAMs were cut with a razor blade and then fixed in a solution composed of acetic acid, ethanol, and chloroform (6:1:3) with iron traces.

Samples were immediately stored at −20 °C for 24 h. After that, the fixing solution was renewed (no iron traces were added this time), and the samples were stored at −20 °C for four days. After fixing, to allow cells and chromosomes dispersion, samples were hydrolyzed at 60 °C for 20 min in HCl 1N. Then, to stain the chromosomes, RAM samples were immersed in Schiff reagent and placed under dark conditions for 2 h at room temperature. Finally, the reaction was stopped with a few drops of acetic acid, and the samples were mounted on a microscope slide and fixed, passing the mounted slide on a flame for 3 s. A total of 1000 cells were counted in between 30 and 40 meristems per treatment. Samples were observed using an Olympus BX41 optical microscope (100 × objective).

#### 4.4.2. Localization of GFP Signal in Arabidosis’s Primary Roots

The experiments on *pDR5::GFP*, *pPINs::GFP* were carried out on 4 days old seedlings transplanted for 48 h on agar medium enriched with coumarin (short-term experiment). Treated and untreated *A. thaliana* (Col-0) seedlings were collected, fixed, and mounted as previously described [40].

Confocal images of median longitudinal sections were obtained using a Leica inverted TCS SP8 confocal scanning laser microscope equipped with a 40× oil immersion objective. The detection of green fluorescent protein (GFP) (excitation peak centered at about 488 nm and the emission peak wavelength of 509 nm) was performed by combining the microscope’s sequential scanning facility’s settings. More than 40 seedlings were analyzed per treatment, and five independent experiments were carried out. Channel settings for GFP and laser power were kept identical during the analysis of all samples to make the results comparable.

To evaluate auxin and PIN protein abundance, the GFP signal intensity was measured by quantifying the integrated optical density (IOD) parameter on the root tip and maturation zone, using the software ImagePro Plus (Media Cybernetics, Inc. 1700 Rockville Pike, Suite 240 Rockville, Rockville, MD, USA). The measurements have been obtained using the “Count/Size” tool. First, all the green signals in the area of interest have been manually selected using the tool “Intensity Range Selection.” Successively, the IOD of the entire green selected area has been measured on the root tip and maturation zone for the *pDR5::GFP* transgenic line and on the entire root apex for the *PINs*.

#### 4.4.3. Auxin Quantification

Auxin quantification was carried out on *Arabidosis* seedlings treated with coumarin (0 or 100 µM) for 48 h. The entire root of control or treated seedlings was excised, immediately snap-frozen in liquid nitrogen (to quench the metabolism), and powdered. One hundred milligrams of plant material was used for sample extraction and derivatization. Samples were derivatized and analyzed using a GC-MS apparatus following the protocol described by Rawlinson et al. [89] with some modifications.

To the powdered samples, a 20 µL solution of 3-indolepropionic acid (IPA 20 mg/mL) was added as the internal standard for relative quantification and normalization purposes. Successively, 147 µL of methanol (MeOH), 34 µL of pyridine, and 200 µL of NaOH (1% *w*/*v*) were added, and the samples were shaken for 40 s. After extraction, samples were derivatized using 20 µL of methyl chloroformate and shaking for 30 s (this step was repeated two times). To the derivatized samples, 400 µL of chloroform and 400 µL of a NaHCO_3_ solution (50 mM of stock) were added, and the samples were shaken again for 1 min and centrifuged at 14,000 rpm per 1 min. An aliquot (100 µL) of the organic lower phase was collected and used for gas chromatography-mass spectrometry (GC-MS) analysis. A parallel experiment was carried out using, as an external standard, pure IAA for the assignment of the retention time.

GC-MS analysis was carried out using a Thermo Fisher gas chromatograph apparatus (Trace 1310) equipped with a single quadrupole mass spectrometer (ISQ LT) (Thermo Fisher Scientific, Str. Rivoltana, Km 4, 20090 Rodano, Milan, Italy). The capillary column (MEGA-5MS 30 m × 0.25 mm × 0.25 µm + 10 m pre-column) and the carrier gas was helium with a flow rate of 1 mL/min. The injector and transfer line were set at 250 °C and 270 °C, respectively. Samples (3 µL) were injected with a 35 psi pressure pulse, which was held for 1 min. The following GC temperature program was used: Isocratic for 1 min at 40 °C, from 40 °C to 320 °C at a rate of 20 °C min^−1^, and then held isocratic for 2 min at 320 °C. The ion source was set to 200 °C, and the solvent delay was 4.5 min. Mass spectra were recorded in electronic ionization (EI) mode at 70 eV, scanning at a 50–400 m/z range to select appropriate EI mass fragments for each analyte. Then, the MS was run in selected ion monitoring (SIM) using one quantifier (m/z) and two qualifiers (m/z) ions. In particular, for IAA-methyl ester, the ions 189, 103, and 77 were selected for quantification. IAA identification and quantification were performed by comparing the RT with the IAA external standard and the mass spectra in the National Institute Standard and Technology (NIST 2011) spectral library. The relative IAA quantification was carried out by normalizing the IAA peak intensity with the intensity of the internal standard.

### 4.5. Statistical Analysis

A completely random design with 20 replications (4 for IAA quantification and 10 for the measurement of the GFP signal intensity) was adopted. Data were first checked for deviations from normality (D’Agostino-Pearson test) and tested for homogeneity (Leven Median test). The significance of differences between data sets was evaluated by the Student’s test (*p* ≤ 0.05).

All anatomical measurements were carried out using the open-source software ImageJ.

## 5. Conclusions

The results suggest that coumarin strongly affects the RAM morphology of *Arabidopsis thaliana*, altering the microtubule cortical array organization, and unbiasing auxin biosynthesis and distribution. Coumarin, as also reported for other microtubule de-polymerizing drugs (e.g., oryzalin), through the alteration in IAA efflux carriers distribution, altered the normal acropetal transport of auxin from the maturation zone to the apical RAM, which was guaranteed only by PIN1 and PIN7. Consequently, it resulted in an auxin accumulation in the maturation zone’s pericycle cell, inducing lateral root formation.

Further studies will focus on understanding whether coumarin acts as a microtubule polymerizing or depolymerizing agent, as well as on studying the signaling involved in response to coumarin using mutants characterized by cyclin and microtubule alterations. Moreover, we will study coumarin’s effects through combined-*omics* approaches.

## Data Availability

Not applicable.

## Supplementary Materials

The following are available online at https://www.mdpi.com/article/10.3390/ijms22147305/s1.
