# Supplementary material for: Coumarin Interferes with Polar Auxin Transport Altering Microtubule Cortical Array Organization in Arabidopsis thaliana (L.) Heynh. Root Apical Meristem
Source: Int J Mol Sci. 2021 Jul 7;22(14):7305. doi: 10.3390/ijms22147305 (PMC8306912; doi:10.3390/ijms22147305)
Supplement: Supplementary file 1 [file ijms-22-07305-s001.zip › ijms-1278728-supplementary.pdf]

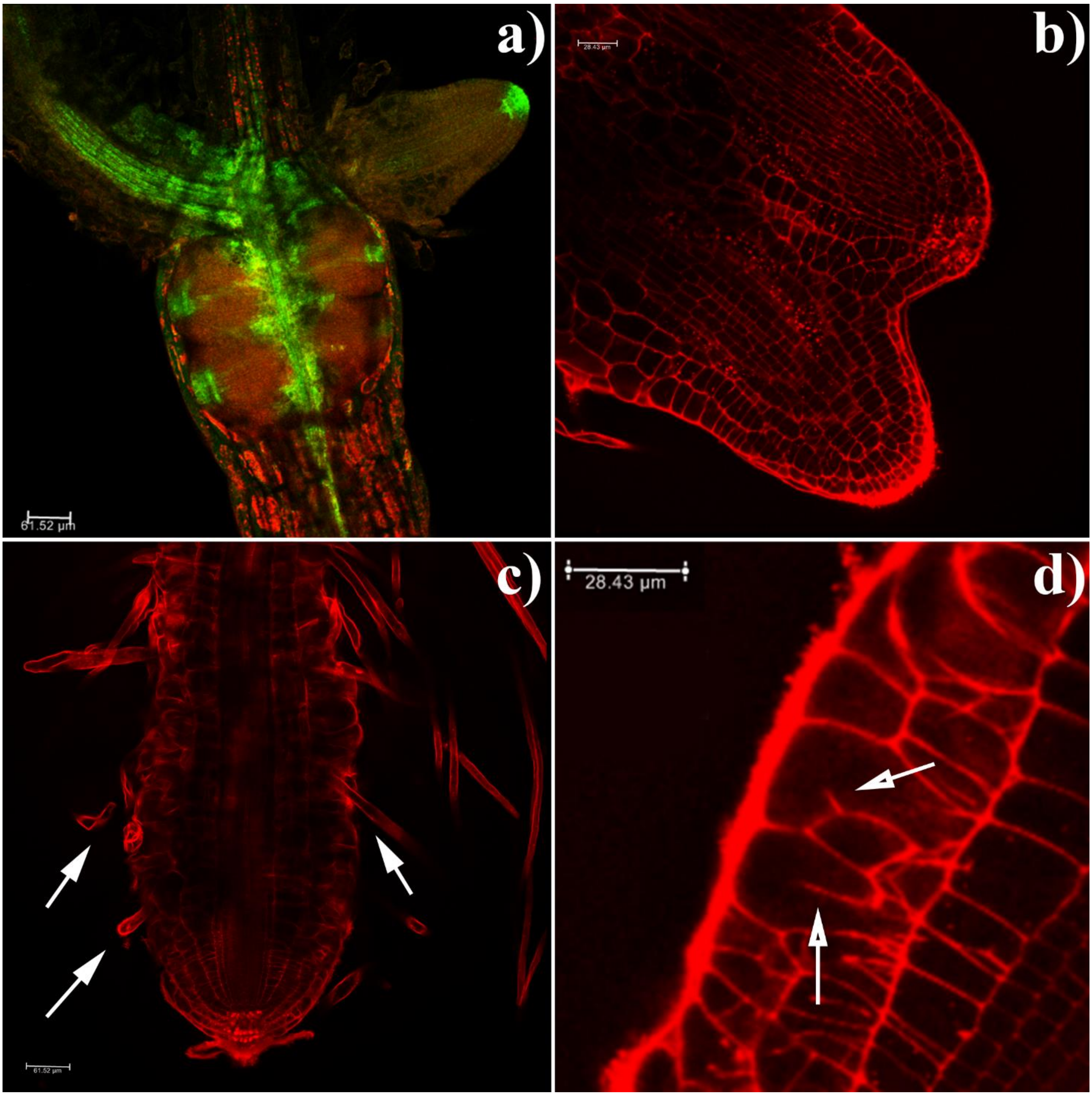

Figure S1. Alterations observed in 4 days old Arabidopsis roots treated with coumarin for 7 days: (**a**) increment in adventitious root production; (**b**) fused adventitious roots; (**c**) stocky meristem with formation of root hairs (white arrows) close to the meristem; (**d**) incomplete cell division.

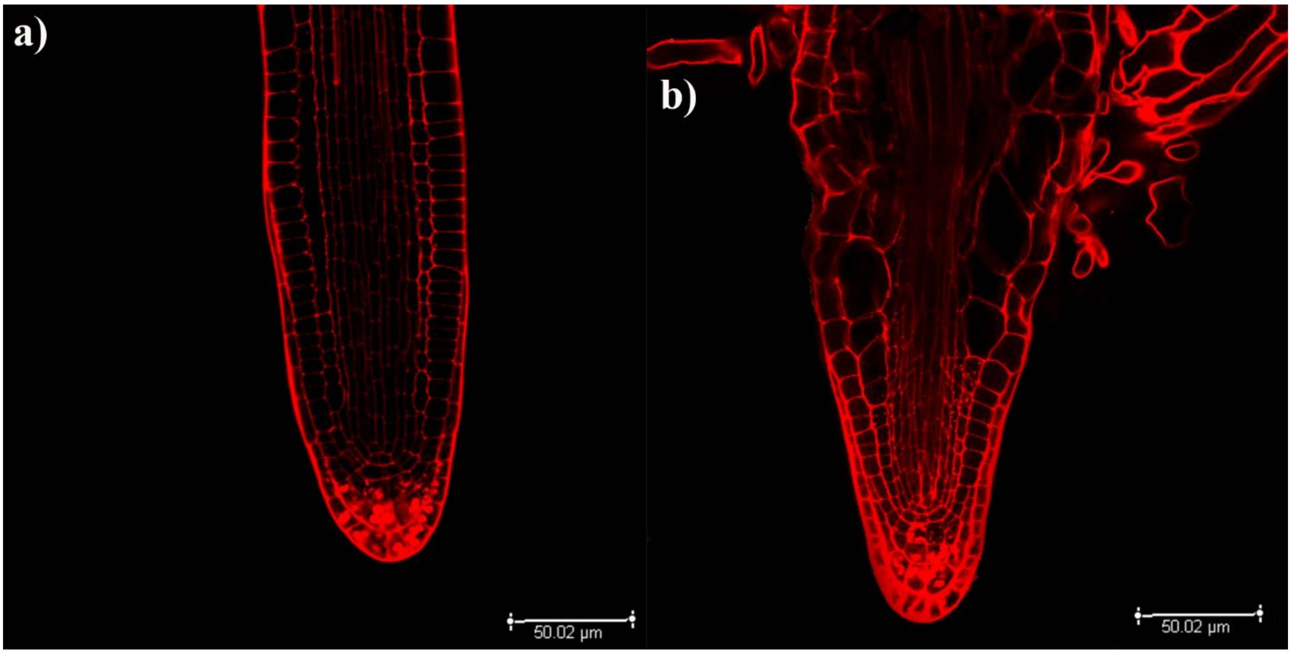

Figure S2. Alterations observed in 4 days old Arabidopsis roots treated with colchicine (200 µM) for 7 days: (**a**) control root; (**b**) treated root. Scale bars 50.02 µm. N = 20.

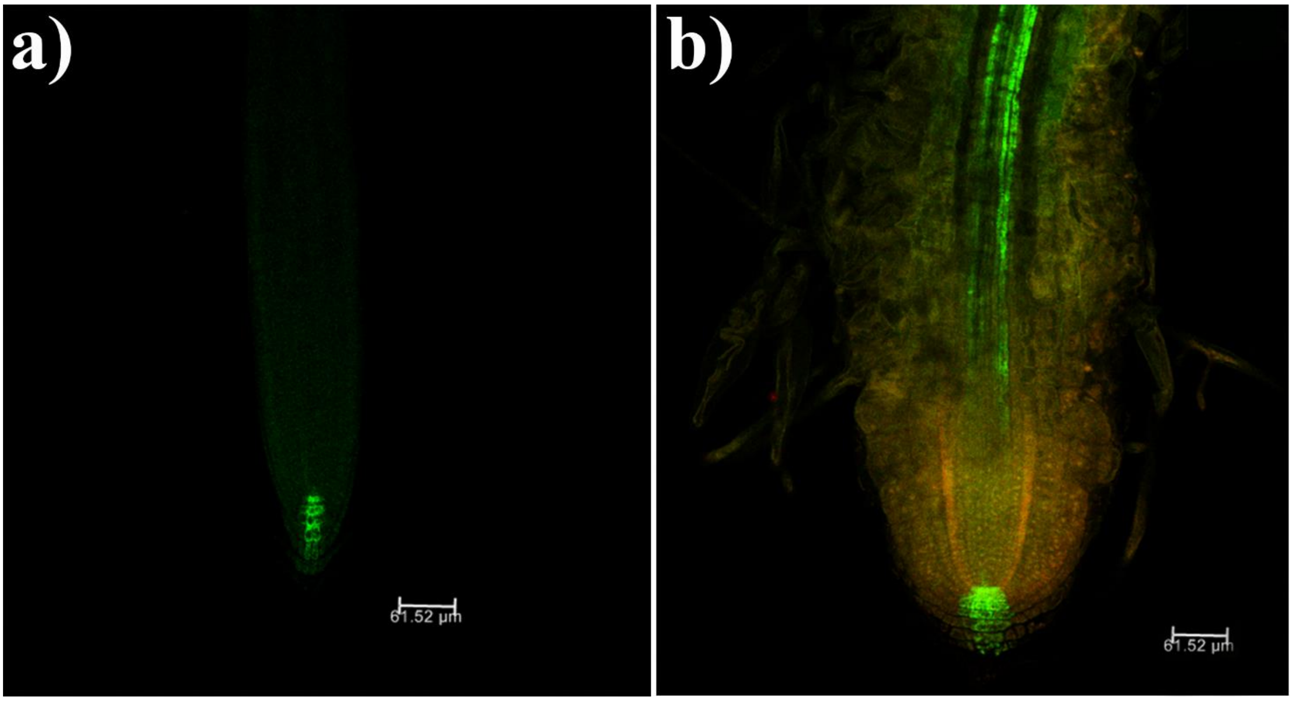

Figure S3. Fluorescence detection in 4 days old *A. thaliana pDR5::GFP* transgenic line (auxin responsive reporter) grown for 7 days in untreated agar medium (**a**) and coumarin 100 µM enriched agar medium (**b**). Scale bars 61.52 µm. N = 20.
